# Functional consequence of the p53 codon 72 polymorphism in colorectal cancer

**DOI:** 10.18632/oncotarget.20580

**Published:** 2017-08-29

**Authors:** Venkat R. Katkoori, Upender Manne, Lakshmi S. Chaturvedi, Marc D. Basson, Pam Haan, Daniel Coffey, Harvey L. Bumpers

**Affiliations:** ^1^ Department of Surgery, Michigan State University, College of Human Medicine, Lansing, MI, USA; ^2^ Department of Pathology, University of Alabama at Birmingham, Birmingham, AL, USA; ^3^ Department of Surgery, University of North Dakota, School of Medicine and Health Sciences, Grand Forks, ND, USA; ^4^ Capital Colorectal Surgery, Lansing, MI, USA

**Keywords:** p53, codon 72 polymorphism, p38MAPK, EMT, CREB

## Abstract

**Background:**

The codon 72 polymorphism in p53 has been implicated in colorectal cancer (CRC) risk, prognosis and CRC health disparities. We examined the functional consequence of this polymorphism in CRC.

**Experimental Design:**

Plasmids (pCMV6) that express different phenotypes of p53 [p53 wild type (wt) at codon 72 (R72^wt^), R72^wt^ with mutation at codon 273 cysteine (R72^273Cys^), p53 mutation at codon 72 (P72^wt^) and P72^wt^ with mutation at codon 273 (P72^273Cys^)] were constructed. The CRC cell line Caco2, which does not express p53 for *in vitro* studies, was used as host. CRC xenografts were established in severe combined immunodeficient (SCID) mice using established cell lines. CRC surgical specimens, corresponding normal colon, and tumor xenografts were sequenced for codon 72 polymorphism of p53. Proteins signaling mechanisms were evaluated to assess the functional consequence of P72 phenotype of p53.

**Results:**

This study demonstrated a significantly increased survival of cells expressing P72^wt^, mutant phenotype, versus R72^wt^ phenotype. WB analyses revealed that P72^wt^ induced activation of p38 and RAF/MEK/ extracellular signal-regulated kinase (ERK) MAP kinases. Activation of CREB was found to be higher in tumors that exhibit P72 phenotype. Metastatic lesions of CRC expressed more phospho-CREB than non-metastatic lesions. The expression of P72^wt^ promoted CRC metastasis.

**Conclusions:**

P72 contributes to the aggressiveness of CRC. Because P72 is over-expressed in CRC, specifically in African-American patients, this suggests a role for P72 in cancer health disparities. This work was supported by NIH/NCI Workforce Diversity Grant R21-CA171251 & U54CA118948.

## INTRODUCTION

Colorectal cancer (CRC) is believed to develop through the accumulation of genetic alterations that deregulate cell growth [1–3]. Sporadic and germline *p53* mutations have been detected in multiple solid tumors including CRC [4]. However, only a subset of these mutant alleles has been proven to cause tumor progression and poor clinical outcomes [5, 6]. Missense point mutations that disrupt *p53* functional domains result in its inactivation and are associated with chemoresistance and poor patient survival [7].

Germline mutations (single nucleotide polymorphisms [SNP]) have been reported in the coding region [8–11] and also in the intronic regions of *p53* [12–20] with cancer risk. Among all these, codon 47 and 72 polymorphisms of p53 have been well characterized [9, 21]. The codon 72 polymorphism is a common alteration in the general population that results in either an arginine (R72) or a proline (P72) residue at position 72 in the proline-rich domain (residues 64–92) of the p53 protein, resulting in a marked change in its protein structure [10]. It has been reported that this region is required for the growth suppression and apoptosis mediated by p53 [22, 23].

Previous studies, *in vivo* and *in vitro*, have highlighted the functional difference between the P72 and R72 variants [21–24] of p53 such as different binding to components of the transcriptional machinery and different activation of transcription, but they did not differ in their ability to bind DNA. It has been reported that there was a significant association between the P72 and risk of cancer [25, 26], although the results with regard to most cancer diseases, including CRC remain inconclusive.

P72 was found to be preferentially mutated and associated with poor prognosis of CRC [26–29]. Furthermore, when compared with other known p53 polymorphisms, the P72 exhibits a higher level of frequency and correlates with cancer progression [30, 31]. However, its mechanistic role in tumor progression is unknown. Therefore, in this study, we evaluated the functional role of this frequently occurring polymorphism in CRC.

## RESULTS

### P72 phenotype of p53 induces cell survival and endothelial cell tubular formation in CRC

**Figure 1:**
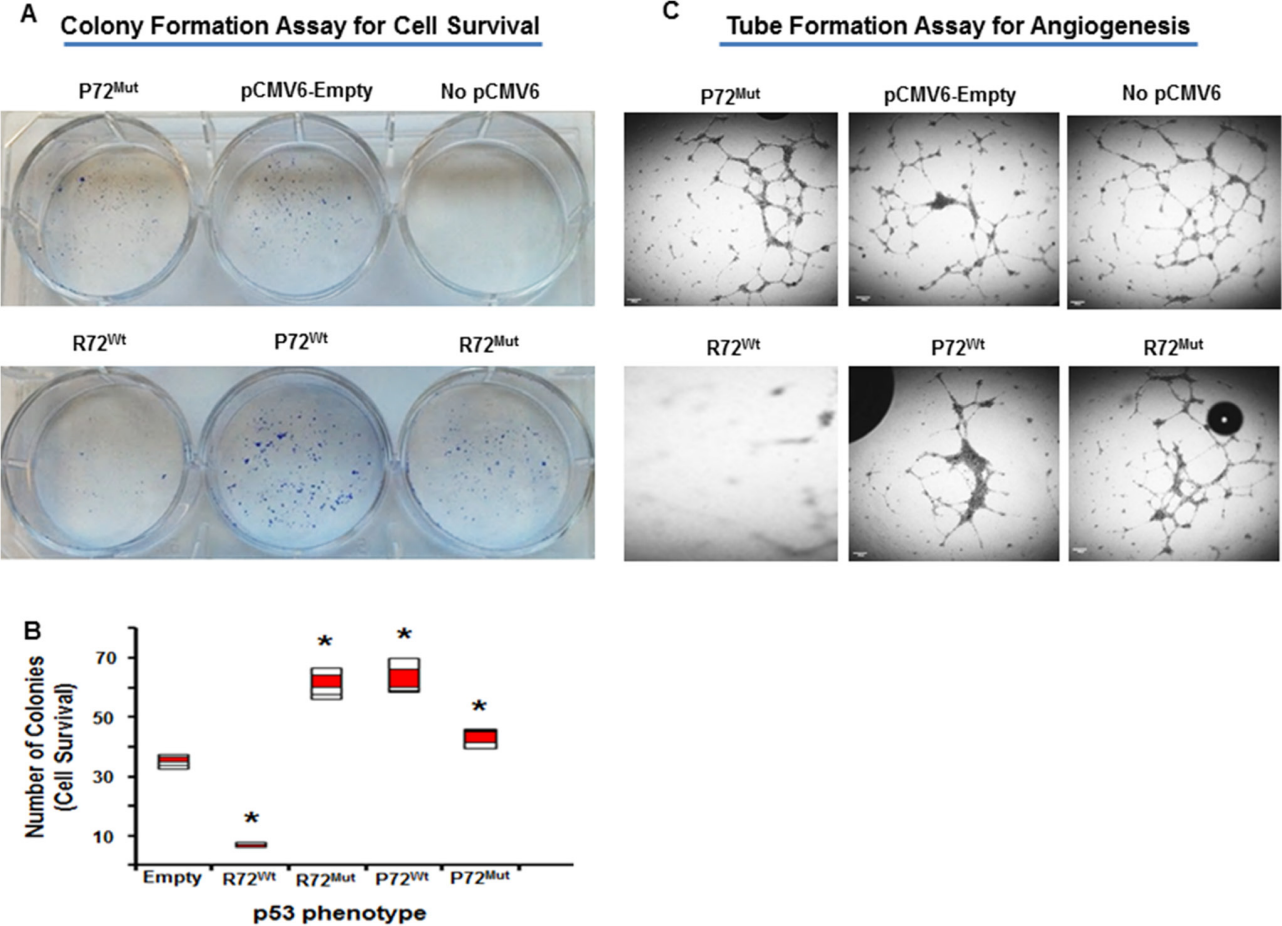
P72 phenotype of p53 induces cell survival and endothelial cell tubular formation Increased cell survival in cells that express P72^wt^ phenotype or mutant phenotypes compared to cells that express R72^wt^ phenotype (**A** and **B**). Conditioned medium of P72^wt^ or mutant phenotypes expressing cells induce HUVEC to form networks of multiple tube-like structures, while conditioned medium of R72^wt^ expressing cells did not support tubule formation (**C**).

This study demonstrated that there was a significant increased cell survival in cells that express P72^wt^ phenotype (p,.02) or mutant phenotypes (*p* < .05) compared to cells that express R72^wt^ phenotype (Figure 1A and 1B). The P72^wt^ or mutant phenotypes induce Human umbilical vein endothelial cells (HUVEC) to form networks of multiple tube-like structures, while conditioned medium from R72^wt^ expressing Caco2 cells did not support tubular formation (Figure 1C). Morphological changes in Caco2 cells were analyzed at 12 h after transfection, and R72^wt^ expressing Caco2 cells exhibited cellular shrinkage and irregular shape or growth suppression (data not shown).

### P72 phenotype is associated with abundance of tumor promoting phenotypes in CRC

**Figure 2:**
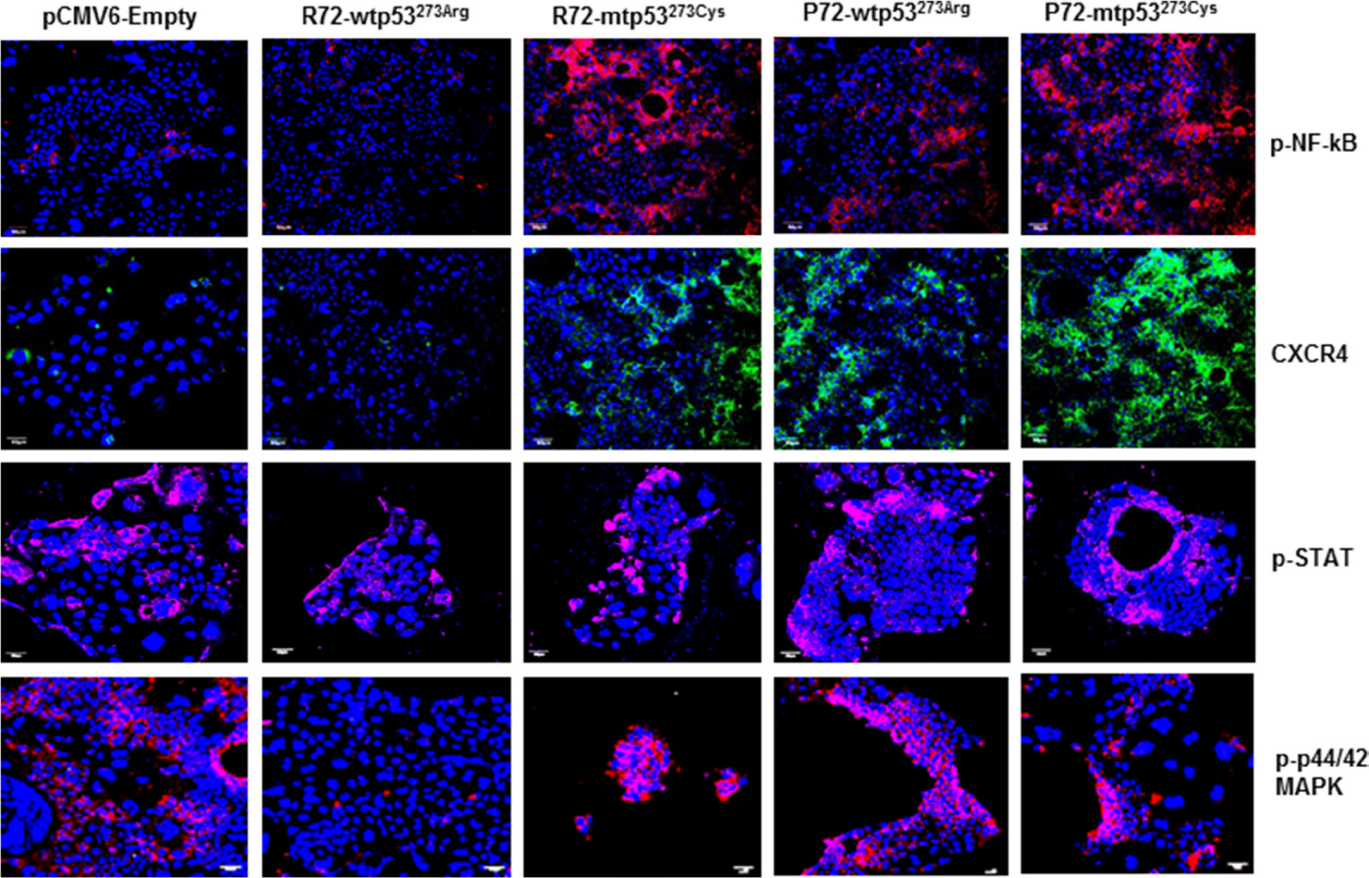
P72 phenotype of p53 is associated with abundance of tumor promoting phenotypes The expression of p-NF-kB, p-STAT, CXCR4 and p-ERK was found to be higher in the cell model of P72^wt^ or mutant phenotypes than in the vector-control or R72^wt^ cell models.

Based on our recent findings that P72 expression in CRC has been found to be associated with CRC aggressiveness [29], we hypothesized that P72^wt^ could induce expression of tumor promoting phenotypes in CRC. To evaluate expression of these phenotypes in cell based model, we analyzed the expression of the commonly used tumor markers phosphorylated NF-kB (p-NF-kB), phosphorylated STAT (p-STAT), CXCR4 and phosphorylated extracellular signal-regulated kinase (p-ERK) MAP kinase, by immunofluorescence. As shown in Figure 2, the expression of p-NF-kB, p-STAT, CXCR4 and p-ERK MAP kinase was found to be higher in the P72^wt^ or mutant phenotypes cell model than in the vector-control or R72^wt^ cell model.

### P72 phenotype promotes EMT process in CRC

**Figure 3:**
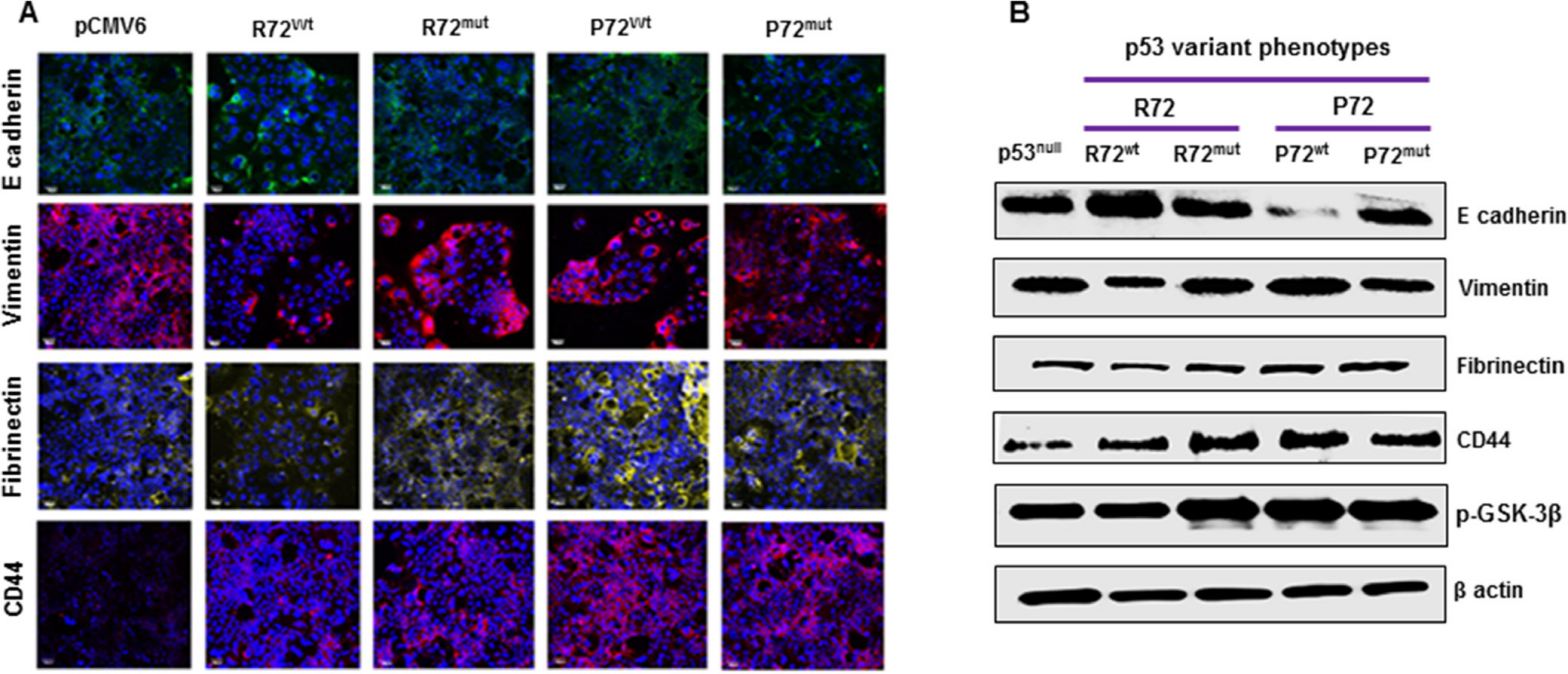
P72 phenotype promotes EMT process (**A** and **B**). P72^wt^ or mutant phenotype expressing cells displayed decreased expression of the epithelial signature E-cadherin and increased expression of the mesenchymal signature vimentin, fibrinectin, CD44 and p-GSK-3β.

We next evaluated the effect of P72 phenotype on the development of EMT process. EMT is a phenotypic conversion that facilitates development of neoplasia, and is associated with tumor progression and metastasis [32]. During this process E-cadherin (Epithelial marker) is down regulated and vimentin, fibronectin, CD44 and phosphorylated glycogen synthase kinase-3β [p-GSK-3 β] (Mesenchymal markers) are up regulated. P72^wt^ or mutant phenotype expressing cells analyzed via western blot analysis displayed decreased expression of the epithelial signature E-cadherin and increased expression of the mesenchymal signature vimentin, fibronectin, CD44 and p-GSK-3 β (Figure 3A and 3B). This pattern is more mesenchymal and less epithelial in nature. These results indicate that P72^wt^ or mutant phenotypes could induce tumor progression through promoting the EMT process.

### P72 phenotype promotes MAP kinase signaling pathway in CRC

**Figure 4:**
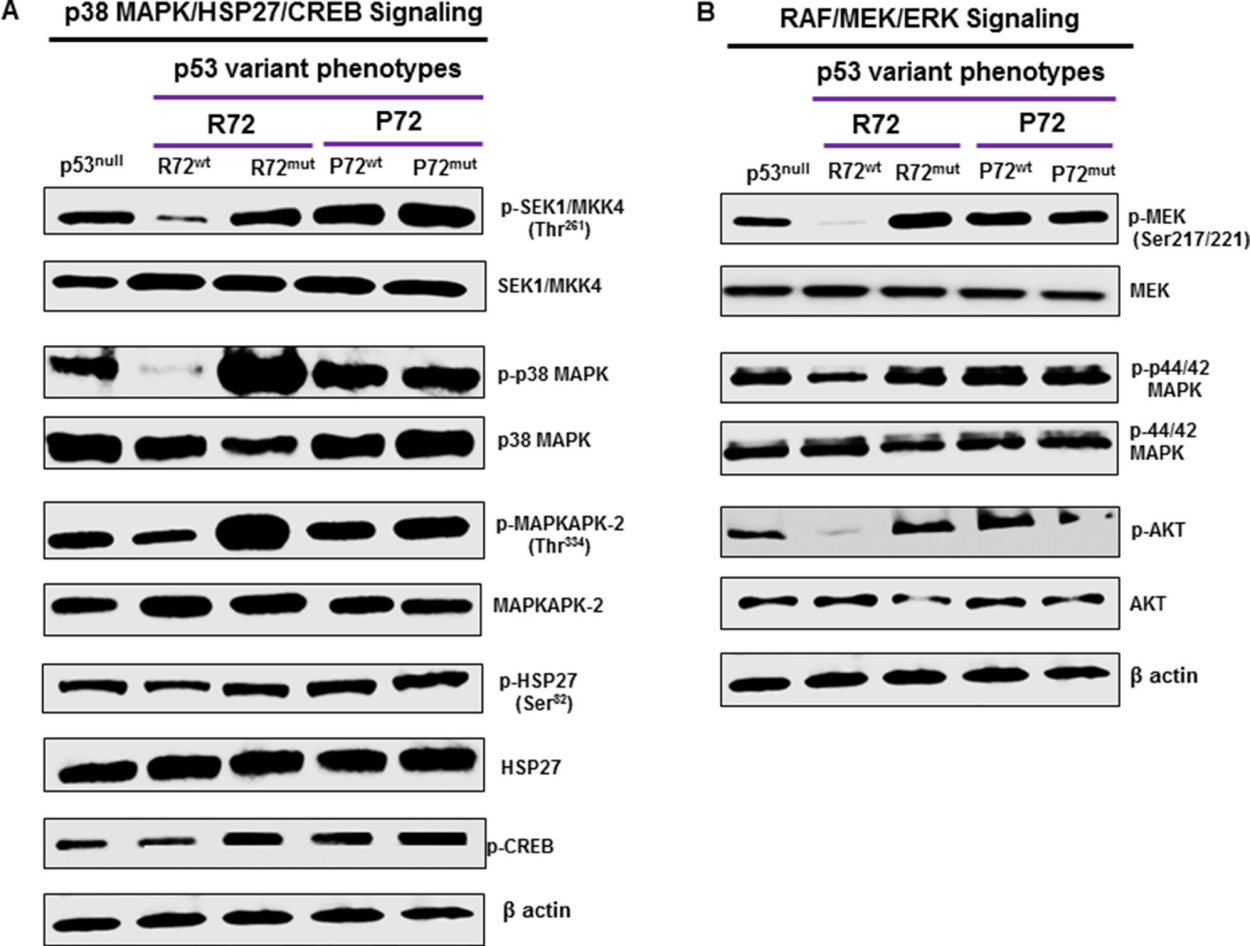
P72 phenotype promotes p38MAPK and RAF/MEK/ERK signaling Molecular analyses revealed that P72^wt^ or mutant phenotypes effectively induced the activation of p38 MAPK and RAF/MEK/ERK signaling (**A** and **B**). Up-regulation of phosphorylated SEK1/MKK4, upstream kinase of p38 MAPK has been found to be associated with P72^wt^ or mutant phenotypes (A). Activation SEK1/MKK4 was accompanied by up-regulation of p-MAPKAPK-2, p-Hsp27 and p-CREB downstream targets of p38 MAPK (A). Suppression of RAF/MEK/ERK activation was significantly higher in cells that express P72^wt^ or mutant phenotypes compared to cells that express R72^wt^ phenotype. This suppression was accompanied by down-regulation of p-Akt (B).

The MAP kinase signaling is frequently activated in several cancers including CRC [33]. The activation of MAP kinase and the subsequent phosphorylation and activation of its downstream targets play an important role in promoting cell growth, proliferation and metastasis in cancer [34]. Molecular analyses revealed that P72^wt^ or mutant phenotypes effectively induced the activation of p38 MAP kinase pathway. Up-regulation of phosphorylated SEK1/MKK4, upstream kinase of p38 MAPK (Figure 4A) has been found to be associated with P72^wt^ or mutant phenotypes. Activation SEK1/MKK4 was accompanied by up-regulation of phosphorylated-MAPKAPK-2 (p-MAPKAPK-2) and phosphorylated-Hsp27 (p-HSP27), and phosphorylated-CREB (p-CREB) downstream targets of p38 MAPK (Figure 4A).

### P72 phenotype promotes RAF/MEK/ERK signaling pathway in CRC

Raf kinases are well characterized as key regulators of the MEK/ERK pathway [35] and activated through the RAF/MEK/ERK pathway. This pathway has an important role in cancer progression [36]. Activation of key proteins in the RAF/MEK/ERK pathway was determined by western blot analysis to assess the effect of P72^wt^ or mutant phenotypes of p53 on this signaling pathway in CRC cells. Suppression of RAF/MEK/ERK activation was significantly higher in cells that express R72^wt^ phenotype compared to cells that express P72^wt^ or mutant phenotypes. This suppression was accompanied by down-regulation of phosphorylated Akt [p-Akt] (Figure 4B), a downstream target of RAF/MEK/ERK.

### High frequency of codon 72 polymorphism of p53 in CRC

Analysis of p53 at codon 72 for the status of variant forms by genotyping revealed a higher frequency of Arg/Pro phenotypes (18/34, 53%) than Arg/Arg (9 of 34, 26%) or Pro/Pro (7 of 34, 21%) in these CRC; however, in this study homozygous Pro/Pro was observed more frequency compared to other previous studies (data not shown). Sequencing analysis of p53 at codon 72 for the status of variant forms by genotyping revealed that SW480 and WiDr tumors demonstrated homozygous for Pro/Pro, whereas HT29 and LS174T tumors demonstrated homozygous for Arg/Arg. The correlation of p53 codon 72 polymorphism statuses in cell line derived tumors based on molecular or clinical features is shown in Tables 1 and 2.

**Table 1:** Genetic or clinical features of 4 CRC cell lines

Sample ID	p53codon 72	P53 Mut	MSI St	cMyc	KRAS	CIN	Clinical Stage	Race
SW480	Pro/Pro	R273H	MSS	+	G12V	+	II B	Caucasian
WiDr	Pro/Pro	R273H	MSS	–a	Wt	+	–a	-
LS174T	Arg/Arg	Wt	MSI	+	G12D	-	IIB	Caucasian
HT29	Arg/Arg	R273H	MSS	+	Wt	+	–a	Caucasian

Abbreviations: Codon 72, p53 phenotype of codon 72 polymorphism; Pro/Pro, homozygous for proline phenotype at codon 72 of p53; Arg/Arg, homozygous for arginine phenotype at codon 72 of p53; p53 Mut, p53 mutational status. Wt, p53 wild type; MSI St, microsatellite instability status; MSS, microsatellite stable; MSI, microsatellite instability; –a, No publication on WiDr information on cMyc and clinical stage was reported; However WiDr and HT 29 are identical cell lines although status of codon 72 of p53 is different; CIN, chromosomal instability pathway. Genetic or clinical features of all these cell lines obtained from published source.

**Table 2:** Phenotypic expression of different markers in 4 CRC cell lines

Sample ID	p53codon 72	P53 Mut	WAF1%	MDM2%	BAX%	V14_3_3_s	AIP%	GADD45%	NOXA%	P53R2%
SW480	Pro/Pro	R273H/P309S	43.57	103.74	180.39	179.76	136.41	97.42	182.03	165.94
WiDr	Pro/Pro	R273H	1.01	0.00	2.42	0.00	0.00	0.00	0.00	15.97
LS174T	Arg/Arg	Wt	–a	–a	–a	–a	–a	–a	–a	–a
HT29	Arg/Arg	R273H	1.01	0.00	2.42	0.00	0.00	0.00	0.00	15.97

Abbreviations: Codon 72, p53 phenotype of codon 72 polymorphism; Pro/Pro, homozygous for proline phenotype at codon 72 of p53; Arg/Arg, homozygous for arginine phenotype at codon 72 of p53; p53 Mut, p53 mutational status. Wt, p53 wild type; –a, No publication on LS174T information on phenotype was reported; However WiDr and HT 29 are identical cell lines although status of codon 72 of p53 is different. Phenotypic expression of different markers for all these cell lines obtained from published source.

### CREB is progressively expressed in advanced disease

**Figure 5:**
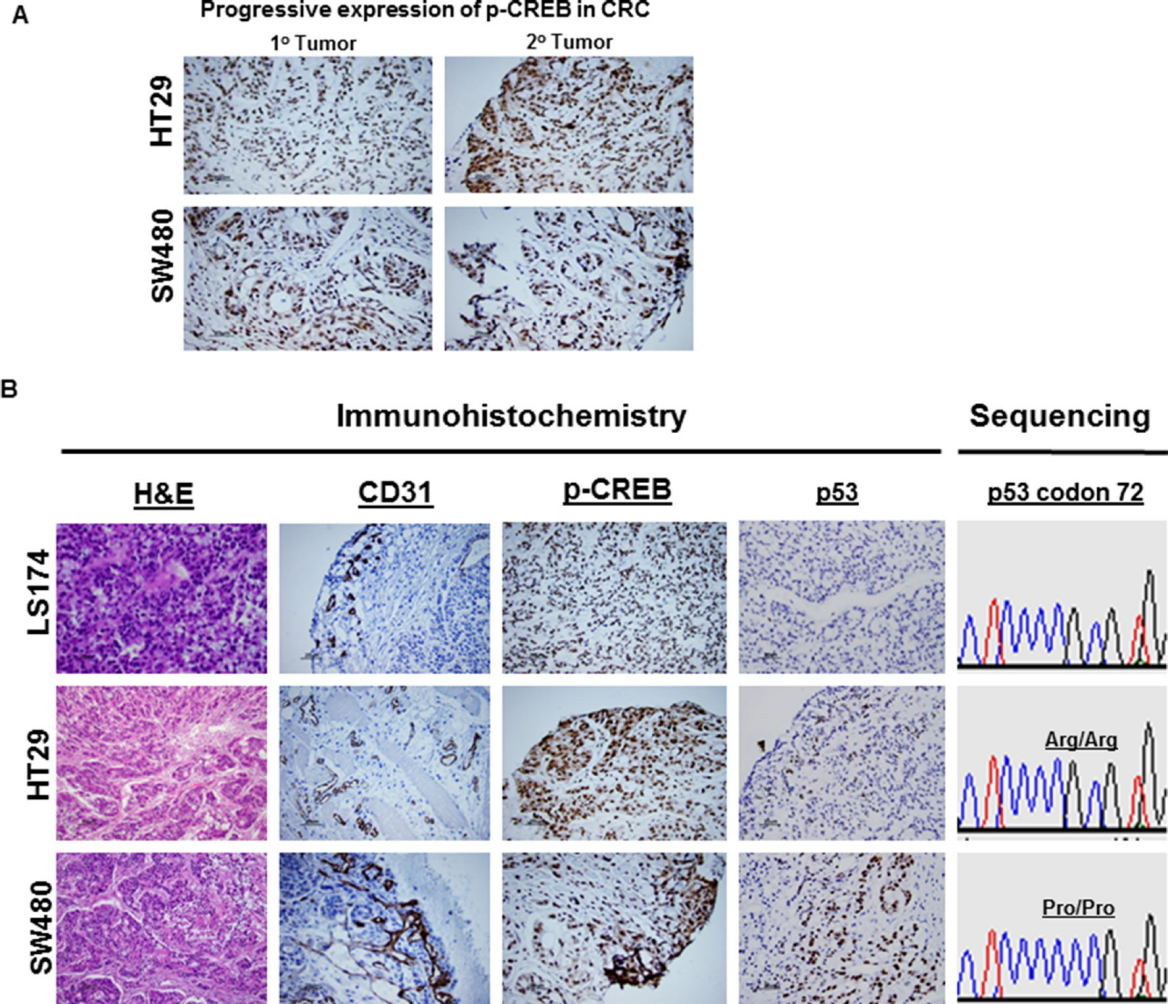
CREB expression in tumor xenografts by IHC IHC confirmed the presence of CREB expression in CRC (HT29) and (SW480) (**A**). Compared to primary CRC lesion, the expression of CREB was markedly increased in metastatic CRC lesions (A). IHC confirmed the expression status of CD31, CREB, and p53 in CRC (HT29) and (SW480) (**B**). Compared to R72 CRC, the expression of CD31 (tumor angiogenesis) or CREB was markedly increased in P72 CRC. Sequencing analysis revealed that LS174T or HT29 tumors exhibit homozygous for Arg/Arg, whereas SW480 tumor exhibits homozygous for Pro/Pro (B). IHC analysis revealed that LS174T tumor was found to be negative for p53 expression (p53 wild type) (B), whereas HT29 or SW480 tumors were found to be positive for p53 expression (p53 mutants) (B).

**Figure 6:**
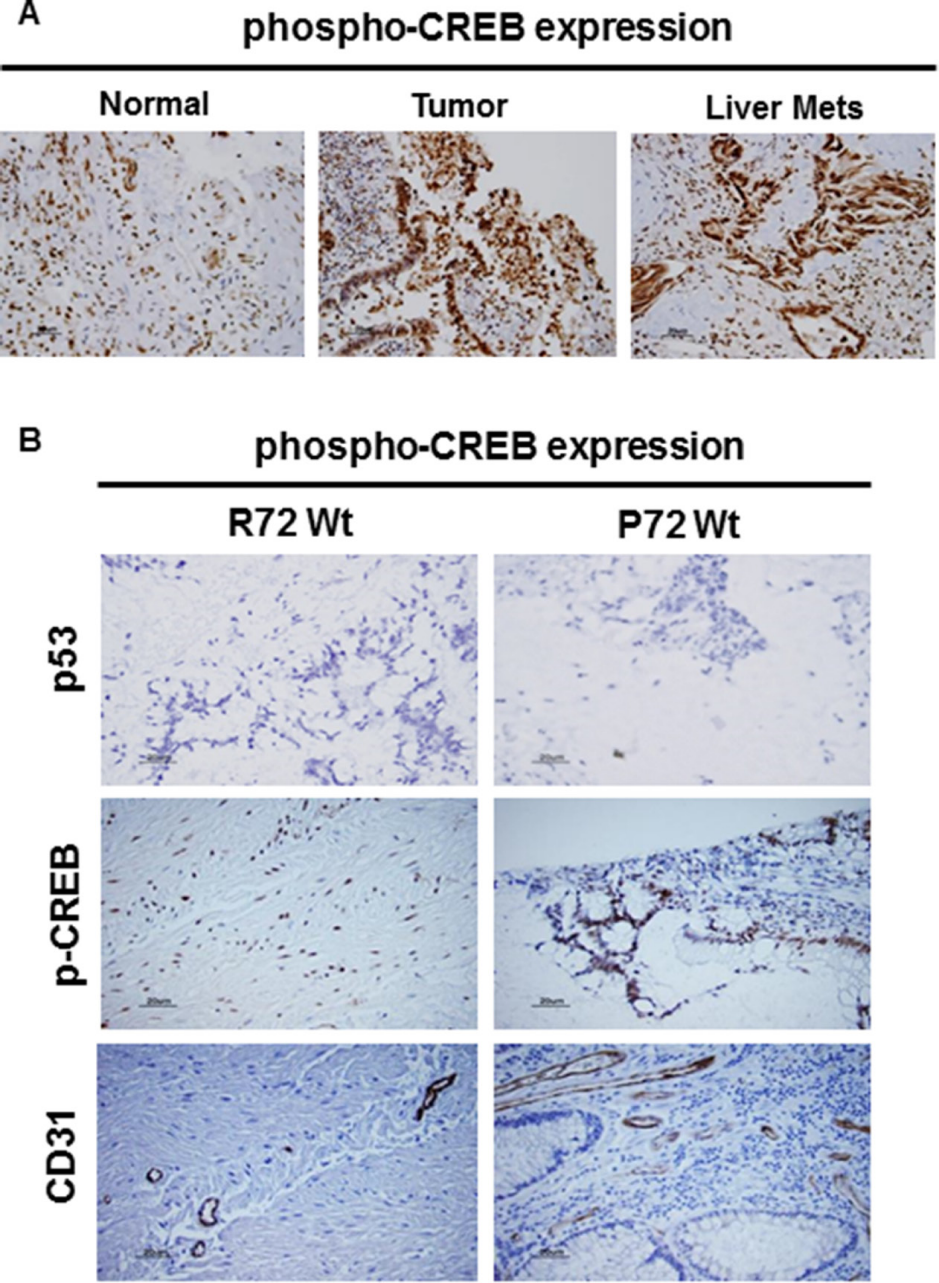
CREB expression in CRC by IHC Immunostaining confirmed the presence of CREB expression in CRC (**A**). Compared to normal, the expression of CREB was markedly increased in primary CRC lesion or metastatic CRC lesions (A). (**B**) IHC confirmed the expression status of CD31, CREB, and p53 in CRC (B). Compared to R72 CRC, the expression of CD31 (tumor angiogenesis) or CREB was markedly increased in P72 CRC. IHC analysis revealed that tumors were found to be negative for p53 expression (p53 wild type) (B).

The expression of phospho-CREB in paired primary and metastatic lesions of CRC obtained from xenografts was compared. The expression of phospho-CREB was substantially higher in metastatic tumors than in the corresponding non-metastatic tumors (Figures 5A and 6A). This suggests that phospho-CREB is progressively expressed as malignant disease becomes more advanced, thus there appears to be a direct association with tumor progression and metastasis.

### P72 phenotype promotes activation of CREB and angiogenesis in tumor xenografts and CRC tissues

CREB has been found to be a critical regulator of cell differentiation and involved in tumor progression and prognosis, supporting its role as a proto-oncogene [37] CREB can be activated through phosphorylation by a number of kinases, including Akt, p90Rsk, protein kinase A, and calcium/calmodulin-dependent kinases and controls genes that promotes tumor progression including Egr-1, Bcl-2 family members, and cyclins. Over-activation of CREB was observed in cancer tissues from patients with prostate cancer [38–41], non-small-cell lung cancer [42] and acute leukemia [43], while down-regulation of CREB in several distinct cancer cell lines resulted in inhibition of cell proliferation and induction of apoptosis, suggesting that CREB may be a promising target for cancer therapy [44]. Given the potential roles of CREB in tumorigenesis, tumor xenografts and CRC tissues were then used to study whether P72 phenotype of p53 associated with its activation. We observed that the tumors expressing P72 exhibited increased CREB activation (Figures 5B and 6B). Furthermore, it was also found that tumors with P72 had well established vascularity, while R72 tumors had poor vascularization. Indeed, the mean MVD was higher in tumors from P72 than in R72 tumors (Figures 5B and 6B).

## DISCUSSION

This study demonstrated that there was a significant increased cell survival in cells that express P72^wt^ phenotype or mutant phenotypes compared to cells that express R72^wt^ phenotype. The P72^wt^ or mutant promoted the formation of networks of multiple tube-like structures and well established vascularity. The mean MVD was higher in tumors from P72 than R72. R72^wt^ expressing Caco2 cells exhibited cellular shrinkage and irregular shape or growth suppression. Western blot analyses revealed that P72^wt^ or mutant phenotypes effectively induced the activation of p38 MAPK/HSP/CREB pathway. Tumors expressing P72 exhibited increased CREB activation. The expression of phospho-CREB was substantially higher in metastatic tumors than in the corresponding primary tumors. Furthermore, suppression of RAF/MEK/ERK activation was significantly higher in cells that express R72^wt^ phenotype compared to cells that express P72^wt^ or mutant phenotypes. This suppression was accompanied by down-regulation of phosphorylated Akt [p-Akt]. The expression of P72^wt^ or mutant phenotypes displayed decreased expression of E cadherin (loss of epithelial nature) and/or an increased expression of vimentin, fibrinectin, CD44 and p-GSK-3β (gain of mesenchymal nature).

The *p53* gene, like the Rb gene, is a tumor suppressor gene, and involved in suppressing tumor progression [45]. Mutations in *p53* are found in several cancers including CRC [46, 4], and contribute to the complex network of molecular events leading to development of tumor promoting phenotype [47]. More than 85% of known cancer-related p53 mutations are missense mutations which result in the disruption of p53 conformation [48, 29]. These consequences might contribute to tumor progression and to a poor prognosis. In agreement with these results, the present study shows a significant increased cell survival in cells that express P72^wt^ phenotype or mutant phenotypes compared to cells that express R72^wt^ phenotype. The P72^wt^ or mutant phenotypes induce the formation of networks of multiple tube-like structures related to tumor angiogenesis. Immunostaining analyses revealed that P72^wt^ or mutant phenotypes effectively induced the activation of NF-kB, STAT phenotypes and expression of CXCR4 and CD44 phenotypes. Activation or expression of these phenotypes is the most common event in solid human cancers, including CRC and it is found to be associated with tumor progression and an independent predictor of poor survival in tumors [49–52]. Thus, it is important to explore new drugs that target P72^wt^ or mutant phenotypes which could have a profound impact as a therapeutic agent to suppress tumor progression.

We have shown that P72^wt^ phenotype exhibited an increased incidence of p53 mutations, specifically of the disruptive type, and was associated with nodal metastasis and short overall survival [29]. This suggests that P72 may induce cellular events that favor tumor progression in CRC. The p38MAPK and RAF/MEK/ERK signaling are two of the most important molecular mechanisms that are associated with tumor angiogenesis and cancer cell survival [53, 54]. Increased expression of proteins that are associated with p38MAPK and RAF/MEK/ERK signaling induces tumor viability or metastasis through enhanced proliferation of cells by activating molecular pathways [55], such as accelerating vascularization by activating VEGF [56]. We demonstrate here that the expression of P72^wt^ or mutant phenotypes in CRC cells is associated with increased activation of p38MAPK kinase and RAF/MEK/ERK signaling. Increased expression of p38MAPK and RAF/MEK/ERK signaling correlates with increased tumor angiogenesis, cell survival, and tumor growth rate, which impairs patient survival in diverse cancers [33]. CREB has been found to be a critical regulator of cell differentiation and involved in tumor progression and prognosis, supporting its role as a proto-oncogene [37]. CREB has been found to be activated by a number of kinases, and controls a gene that promotes tumor progression. Activation of CREB has been observed in cancers of prostate [38], breast [39], non-small-cell lung [46] and acute leukemia [43]. It has been shown that down-regulation of CREB in cancer cells resulted in inhibition of cell proliferation and induction of apoptosis, suggesting that CREB may be a promising target for cancer therapy [44]. Since P72^wt^ of p53 is the most commonly expressed phenotype in cancer and correlated with p38 MAPK/HSP27/CREB signaling, targeting of P72^wt^ of p53 by an appropriate therapeutic agent may be a means of controlling the tumor progression.

EMT, the change from an epithelial phenotype into a mesenchymal phenotype, is an important characteristic of cancer stem cells [37, 57, 58]. EMT phenotype has been found to be associated with p53 mutations that demonstrate gain of oncogenic function [59]. The present study demonstrated a significant association between P72^wt^ or mutant phenotypes and induction of EMT. Therefore, P72^wt^ or mutant phenotypes might be a key regulators of the EMT process through which it could activate signals associated with tumor progression. This data strongly suggest that P72^wt^ or mutant phenotypes are potential targets in the inhibition of tumor angiogenesis and oncogenic EMT process in CRC.

Although additional studies will be warranted to elucidate the molecular mechanisms that are associated with P72^wt^ or mutant phenotypes of p53, our results suggest that the P72 polymorphism of p53 in CRC is a gain of function alteration leading to activation of tumor promoting phenotype. The possibility of P72 blockade may be considered in patients with CRC that are unresponsive to conventional treatments. This is the subject of ongoing research in this area that will be further addressed in future communications.

## MATERIALS AND METHODS

### Cell culture, transfection and colony formation assay

CRC cell line Caco2, which does not express p53 was obtained from American Type Culture Collection (ATCC, Manassas, VA, USA). We maintained these cells at 37°C with 8% CO_2_ in Dulbecco Modified Eagle Medium with 4500 mg/L of D-glucose, 4 mM glutamine, 1 mM sodium pyruvate, 100 U/mL of penicillin, 100 μg/mL of streptomycin, 10 μg/mL of transferrin, 10 mM 4-(2-hydroxyethyl)-1-piperazineethanesulfonic acid (pH 7.4), and 3.7 g/L of sodium bicarbonate, supplemented with 10% fetal bovine serum. All studies were performed on the cells within 15 passages. Plasmids (pCMV6) that express different phenotypes of p53 [p53 wild type (wt) at codon 72 (R72^wt^), R72^wt^ with mutation at codon 273 cysteine (R72^273Cys^), p53 mutation at codon 72 (P72^wt^) and P72^wt^ with mutation at codon 273 (P72^273Cys^)] were constructed. Other CRC cell lines also used in this study (Tables 1 and 2).

Caco2 cells were transiently transfected with pCMV control vector or pCMV-p53 wt or mutant phenotypes expressing vector using TurboFect transfection reagent (Fisher Thermo Scientific, Waltham, MA, USA) for the indicated times according to the manufacturer's instructions. Briefly, 1 ×10^5^ cells were seeded into six-well plates containing medium and incubated overnight. For each well, 4 μg DNA (pCMV or pCMV-p53^wt^ or p53^mut^) was mixed with 100 μL of RPMI-1640. The mixture was then combined with a solution of 2 μL of TurboFect transfection reagent (Fisher Thermo Scientific, Waltham, MA, USA). After a 20-min incubation period at room temperature, the mixture was applied to the cells in final volume of 2 ml. After transfection, the expression of p53 phenotypes was confirmed by western blot analysis. Clones resistant to geneticin were assessed for cell survival; colonies were stained with 0.5% crystal violet and counted.

### Tube formation assay

After thawing Matrigel (EMD Millipore, Billerica, MA, USA) on ice the 96-well plate coated with 50 μL Matrigel in each well was then incubated at 37°C for 30-min to allow the Matrigel to polymerize. To examine the effect of p53 phenotype on tumor cell-induced tube formation of HUVECs (ATCC), a conditioned medium was collected from R72^wt^ or mutant phenotypes transfected Caco2 cells as indicated and used as the growth medium for HUVECs. A total of 1 × 10^4^ HUVECs were seeded into each well that had conditioned medium. Cells were then incubated for 8 h to allow the formation of tube-like structures. There are three parameters by which capillary structure formation can be considered: capillary length, number of capillaries or branched tubes.

### Western analysis

After post transfection, Caco2 cells were prepared for western blot by incubating with lysis solution (1.0% Nonidet P-40; 50 mM Tris-HCl; pH 7.5; 20 mM EDTA buffer) (Sigma-Aldrich, St. Louis, MO, USA) at room temperature for 5-min. The lysates were centrifuged for 20-min at 12,000 rpm at 4°C. The supernatants were collected and stored at −70°C. Protein concentrations were determined with the Bradford assay kit (Bio-Rad Laboratories, Hercules, CA, USA). Portions of each sample (20 μl) were separated by SDS-PAGE on a 4–20% Tris-HCl Criterion precast gel (Bio-Rad Laboratories) and electrophoretically transferred to polyvinylidene difluoride (PVDF) membranes (Life Technologies, Grand Island, NY, USA). The membranes were washed in 1× Tris-buffered saline (TBS) for 5-min, and then blocked with 5% nonfat milk in 1× TTBS (1× TBS and 0.1% Tween 20) for 1 h by shaking at room temperature. For detection of expression status of protein in lysates, a rabbit polyclonal anti-human specific antibody was used. This was accomplished by shaking the membranes at 4°C overnight, as directed by the manufacturer, followed by application of horseradish peroxidase (HRP)-conjugated goat anti-rabbit antibody. Protein bands were detected by SuperSignal West Femto Maximum Sensitivity Substrate Reagent (Fisher Scientific, Pittsburgh, PA, USA), followed by exposure on Odyssey Fc Dual-Mode Imaging System (LICOR, Lincoln, NE, USA). Images were scanned into Adobe Photoshop 5.0.2. After detection of specific protein, the blots were stripped and hybridized with a polyclonal rabbit anti-β-actin, then probed with the HRP-conjugated anti-rabbit antibody for normalization. Details of all antibodies used in this study are shown in Table 3.

**Table 3:** Details of primary antibody used in this study

S. No	Antibody	Catalog^#^	Host	Clonality	Company	Dilutions*	Dilutions^¶^
1	CXCR4	NB600-786	Rabbit	Polyclonal	Novus Bio	1:500	1:1000
2	β-actin	4967S	Rabbit	Polyclonal	CST	-	1:1000
3	VEGF-A	Sc-507	Rabbit	Polyclonal	SCB	1:500	1:1000
4	CD31	Ab28364	Rabbit	Polyclonal	abcam	1:200	-
5	E-cadherin	Sc-7870	Rabbit	Polyclonal	SCB	1:200	1:1000
6	Vimentin	3932S	Rabbit	Polyclonal	CST	1:500	1:1000
7	Fibrinectin	F3648	Rabbit	Polyclonal	Sigma-Aldrich	-	1:1000
8	GSK3-3β	9323P	Rabbit	Monoclonal	CST	-	1:1000
9	p53	628202	Mouse	Monoclonal	BioLegend	1:200	1:1000
10	Akt	9271	Rabbit	Polyclonal	CST	1:25	1:1000
11	Erk	9106	Mouse	Monoclonal	CST	1;100	1:1000
12	CREB	9198	Rabbit	Monoclonal	CST	1:200	1:200
13	Hsp27	9709P	Rabbit	Monoclonal	CST	-	1:200
14	Stat3	9145	Rabbit	Monoclonal	CST	1:100	-
15	MapKaPK-2	3007P	Rabbit	Monoclonal	CST	-	1:200
16	SEK1/MKK4	9156	Rabbit	Polyclonal	CST	-	1:1000
17	p38 MAPK	9211	Rabbit	Polyclonal	CST	-	1:1000
18	MEK	9121	Rabbit	Polyclonal	CST	-	1:1000

IHC; Immunohistochemistry, IF; Immunofluorescence, WB; Western Blot, Novus Bio; Novus Biologicals, CST; Cell Signaling Technology, SCB; Santa Cruz Biotechnology, *; Dilutions for IHC/IF, ¶; Dilutions for WB.

### Immunofluorescence

Caco2 cells were prepared by plating cells (1 × 10^5^) on glass slides with poly-D-lysine (Becton Dickinson, Franklin Lakes, NJ, USA) and then allowing cells to attach overnight. After 48 h of post transfection, cells were subsequently washed with 1xPBS and fixed with formalin free Zinc fixative (Becton Dickinson) for 30-min and washed again. Cells were then permeabilized with 1 × PBS containing 0.1% Triton X-100 for 5-min and washed with 1 × PBS. For immunofluorescence analysis, blocking was performed for cells, with 200 μL of serum blocking solution (Zyagen, San Diego, CA, USA) followed by incubation at room temperature for 60-min in a humidified chamber. The slides of the samples were incubated with 200 μL (2 μg/mL) of rabbit polyclonal anti-human specific antibody for overnight at 4^°^C at room temperature. Samples were washed 3 times with 1xPBS for 5-min each then incubated with 200 μL of biotinylated secondary antibody solution (Zyagen) for 30 min at room temperature. After washing with 1xPBS, samples were covered with 200 μL of streptavidin-FITC conjugate solution (Zyagen) and incubated for 30-min at room temperature. Samples were washed 3 times again with 1 × PBS, then counterstained with 4′, 6-Diamidino-2-Phenylindole, Dilactate (DAPI) solution (Zyagen) for 2-min. Samples were washed 3 times with 1 × PBS for 5-min each and covered with coverslip on to slides using anti-fade fluorescent mounting medium (Zyagen). Confocal images were acquired using the Olympus FluoView FV1000 Confocal Laser Scanning Microscope (Olympus America) configured on a fully automated inverted Ix81microscope using a 40× UPLFLN oil (NA1.3) objective. Negative control without primary antibody for each phenotype was used to show its specificity.

### Animals and tumor xenografts

Severe combined immunodeficient female (SCID) mice were purchased from Taconic Farm (Taconic, NY, USA) at four weeks of age and quarantined for one week prior to use. The mice were inoculated with 2-D cultures of CRC (HT29, SW480, WiDr and LS174T) cells to establish primary tumors (tumor xenografts). All food, water, and bedding were sterilized by autoclaving. The mice resided in micro-filtered cages in a room designated for immune compromised mice. On a daily basis the animals were evaluated regarding health status and tumor growth. Body weight, nutritional intake, general activity level, and ruffling of the mice coats were used to determine the health status. All surgical procedures were done under the laminar flow hood and with strictly sterile protocols. A liquid sterilant, exspor (Alcide Co., Norwalk, CT, USA), was used to sanitize the researcher's gloves and the mouse coat at the site of planned surgery. All surgical procedures were conducted in accordance with the guidelines and under approvals of MSU's IACUC. Tumor xenografts were stored in liquid nitrogen and used for molecular analysis based on codon 72 polymorphism of p53.

### Metastasis model

SCID mouse model was used to establish the hepatic metastatic lesion as reported in our earlier version [60]. Hepatic metastasis was produced injecting HT29 and SW480 cells, these lines derived from a primary carcinoma of the colon, into the spleen with the cell count (1 × 10^6^ cells/0.1 cc). Briefly, splenic injections were done by having the spleens extracorporeally injected under direct vision and then replacing the spleen in its usual anatomical location. Livers were removed from mice at necropsy. The number of gross lesions in the liver was counted by use of a magnification lens. These lesions and corresponding primary tumors were used to assess progressive expression status of phospho-CREB.

### CRC tissue procurement and immunocytochemistry

CRC specimens are collected directly from pathology immediately following surgical resection. These specimens are collected under a protocol approved by the IRB of Michigan State University and Morehouse School of Medicine. Sequencing analysis for codon 72 polymorphism of p53 was performed using genomic DNA purified from CRCs and corresponding normal tissue specimens or tumor xenografts of parent CRC cells as previously described [29].

Histological staining and immunocytochemistry (IHC) were performed as previously described [31]. Paraffin blocks for tumors were prepared with standard histopathology methods. Briefly, tumors were collected, washed twice in 1xPBS, and fixed in formalin free Zinc fixative for 30-min, then washed again. These structures were subsequently embedded in paraffin blocks and sectioned at 5 micron thickness. Deparaffinization of tissue sections was performed with xylene followed by rehydration through a series of ethanol solutions (100%, 95%, 80% and 50%). Sections from tumors were evaluated for phenotypic expression of various cancer critical proteins. The technique used for this was IHC, as previously described [31] using antibodies that exhibit high specificity and affinity. In brief, tissue sections, 5-μ thick, from tissue blocks that are representative of benign and malignant tumor components were incubated with specific 1^°^ antibodies for p53, phospho-CREB and CD31, followed by 2^°^ antibodies and 3′, 3-diaminobenzidine to detect the antigen and antibody complex. After IHC staining, the sections were counterstained with hematoxylin. Appropriate positive and negative control slides was included in each staining run and maintained for quality control. IHC staining was evaluated manually by at least two investigators (Drs. Bumpers and Katkoori). Staining was analyzed in the uninvolved mucosa and in the tumor components by assessing ≥ 500 cells. A semiquantitative immunostaining scoring system (ISS) was utilized as previously reported [61–63]. The slides were evaluated under blinded conditions and estimated the proportion of cells stained on a scale of 0 to +3 (< 5% = 0, 5–25% = +1, 25–50% = +2 and > 50% = +3). The scores derived were combined to obtain the mean ISS. Categorization of cases into low- and high-expressers of, p53, phospho-CREB and CD31 (binarization) was accomplished by pooling cases with IHC scores “0 and +1” and “+2 and +3”, respectively. A chi-square test was used to compare the distribution of phenotypic expression of phospho-CREB.

Tumor angiogenesis on tumor xenografts developed from parent CRC cells (HT29, SW480, and LS174T) or sections of patient tissue specimens as previously described [60] was performed based on immunostaining of endothelial marker (CD31). Microvessel density (MVD) was determined by light microscopy in areas of invasive tumor containing the highest numbers of microvessels per area. Individual microvessel counts were made on a 200× field within the areas of most intense tumor neovascularization.

### Statistical analysis

Statistical analysis was performed using the Student's two-tailed *t*-test, for comparisons. Differences were deemed statistically significant at *p* ≤ 0.05.
